# RDGBα, a PtdIns-PtdOH transfer protein, regulates G-protein-coupled PtdIns(4,5)*P*_2_ signalling during *Drosophila* phototransduction

**DOI:** 10.1242/jcs.173476

**Published:** 2015-09-01

**Authors:** Shweta Yadav, Kathryn Garner, Plamen Georgiev, Michelle Li, Evelyn Gomez-Espinosa, Aniruddha Panda, Swarna Mathre, Hanneke Okkenhaug, Shamshad Cockcroft, Padinjat Raghu

**Affiliations:** 1 National Centre for Biological Sciences, TIFR-GKVK Campus, Bellary Road, Bangalore, Karnataka 560065, India; 2 Department of Neuroscience, Physiology and Pharmacology, University College, London WC1E 6JJ, UK; 3 Inositide Laboratory, Babraham Institute, Cambridge CB22 3AT, UK; 4 Manipal University, Madhav Nagar, Manipal, Karnataka 576104, India

**Keywords:** RDGB, Lipid transfer, Phosphoinositide, PITP

## Abstract

Many membrane receptors activate phospholipase C (PLC) during signalling, triggering changes in the levels of several plasma membrane lipids including phosphatidylinositol (PtdIns), phosphatidic acid (PtdOH) and phosphatidylinositol 4,5-bisphosphate [PtdIns(4,5)*P*_2_]. It is widely believed that exchange of lipids between the plasma membrane and endoplasmic reticulum (ER) is required to restore lipid homeostasis during PLC signalling, yet the mechanism remains unresolved. RDGBα (hereafter RDGB) is a multi-domain protein with a PtdIns transfer protein (PITP) domain (RDGB-PITPd). We find that, *in vitro*, the RDGB-PITPd binds and transfers both PtdOH and PtdIns. In *Drosophila* photoreceptors, which experience high rates of PLC activity, RDGB function is essential for phototransduction. We show that binding of PtdIns to RDGB-PITPd is essential for normal phototransduction; however, this property is insufficient to explain the *in vivo* function because another *Drosophila* PITP (encoded by *vib*) that also binds PtdIns cannot rescue the phenotypes of RDGB deletion. In RDGB mutants, PtdIns(4,5)*P*_2_ resynthesis at the plasma membrane following PLC activation is delayed and PtdOH levels elevate. Thus RDGB couples the turnover of both PtdIns and PtdOH, key lipid intermediates during G-protein-coupled PtdIns(4,5)*P*_2_ turnover.

## Abstract (precis)

**Summary:** RDGB regulates PtdIns and PtdIns(4,5)*P*_2_ homeostasis during G-protein-activated phospholipase C signalling in *Drosophila*.

## INTRODUCTION

Eukaryotic cells are composed of membrane bound subcellular compartments each of which has a unique protein and lipid composition that is central to its function. Despite this, when cells respond to external stimuli, the chemical identity of the plasma membrane is altered. Cell surface receptors that transduce ligand binding by activating phospholipase C (PLC) enzymes exemplify this problem. Many clinically important receptors including receptor tyrosine kinases (e.g. EGFR, T-cell receptor) and G-protein-coupled receptors (GPCRs; e.g. muscarinic acetylcholine, metabotropic glutamate receptor) utilise PLC-based signalling pathways. Hence, understanding the regulation of PLC signalling might provide important insights into the biology and treatment of diseases where such receptor signalling is implicated.

When PLC is activated, the plasma membrane experiences changes in lipid composition. PLC hydrolyses phosphatidylinositol 4,5-bisphosphate [PtdIns(4,5)*P*_2_], a key plasma membrane lipid, to generate diacylglycerol (DAG), which is then converted into phosphatidic acid (PtdOH); thus the levels of DAG and PtdOH at the plasma membrane rise. Remarkably, even when cells experience high PLC activity, PtdIns(4,5)*P*_2_ levels at the plasma membrane remain relatively stable (Balla et al., 2008; Hardie et al., 2001; Willars et al., 1998). This is achieved by restoring its levels by the sequential phosphorylation of phosphatidylinositol (PtdIns) at position 4 and 5 by phosphatidylinositol-4-kinase (PI4K) and phosphatidylinositol-4-phosphate-5-kinase (PIP5K) (Balla et al., 2008; Wang et al., 2008). However, in order to do so the plasma membrane must maintain adequate supply of PtdIns that can be used by PI4K. Thus, during PLC signalling, the plasma membrane faces two challenges, namely to replenish the levels of PtdIns as well as remove PtdOH that accumulates downstream of PLC activation.

PtdOH and PtdIns are both lipids, are thus anchored to membranes and are incapable of diffusing to and from the plasma membrane. How do cells control the levels of these two lipids at the plasma membrane? PtdIns is synthesised at the endoplasmic reticulum (ER) by PtdIns synthase. By contrast, PtdOH is generated by the action of DAG kinase (DGK) but can also be converted back into DAG by a type II PtdOH phosphatase (Garcia-Murillas et al., 2006; Kai et al., 1996). PtdOH must also move from the plasma membrane to the ER where it is converted into cytidine diphosphate diacylglycerol (CDP-DAG) by CDP-DAG synthase (CDS). Thus, during PLC signalling, cells need a strategy by which PtdOH can be transported from the plasma membrane to the ER and, conversely, one that allows PtdIns to be transported in the reverse direction, namely from the ER to the plasma membrane (Michell, 1975).

Lipids can be transported to and from the plasma membrane by multiple mechanisms. Vesicular transport is a possibility, although the time scale of such transport is not compatible with the high rate at which PLC utilises PtdIns(4,5)*P*_2_ (Liu et al., 2009; Raghu et al., 2012). An alternative mechanism is the exchange of lipids by lipid transfer proteins, and it has been suggested that this might occur more effectively at membrane contact sites (Chang et al., 2013; Levine, 2004). Several studies have suggested that PtdIns might be transported by PtdIns transfer proteins (PITPs), which are able to transfer PtdIns between membranes *in vitro* (Cockcroft and Carvou, 2007; Helmkamp, 1990; Phillips et al., 2006; Wirtz, 1991). Using cellular reconstitution assays PITPs have been defined as factors required to support sustained receptor-activated PLC signalling in cells (Kauffmann-Zeh et al., 1995; Thomas et al., 1993). PITPs are conserved in eukaryotes (reviewed in Cockcroft and Garner, 2011; Routt and Bankaitis, 2004) with two classes of metazoan PITPs being identified, class I and II. Although all PITPs contain a PITP domain (PITPd), in class IIA PITPs, several additional domains and motifs are present. Class II PITPs are often referred to as RDGB proteins because the founding member of this class is the retinal degeneration B (RDGBα; CG11111, hereafter RDGB) protein in *Drosophila* (Harris and Stark, 1977; Hotta and Benzer, 1970). By contrast there is limited information on PtdOH transfer proteins. In yeast, the protein Ups1 in complex with Mdm35 has been shown to function in PtdOH transfer at mitochondrial membranes (Connerth et al., 2012). Additionally, mammalian RDGBβ (also known as PITPNC1), a member of the class II PITP family, has been shown to bind and transfer PtdOH *in vitro* (Garner et al., 2012).

Although several studies have implicated PITPs in cellular functions including cytokinesis, neurite outgrowth and membrane trafficking (Bankaitis et al., 2005; Carvou et al., 2010; Cosker et al., 2008; Giansanti et al., 2006), the biochemical basis of PITP function *in vivo* remains unclear. Much attention has focussed on the ability of the PITP domain to bind and transfer PtdIns and the importance of this activity in supporting PLC-based signalling *in vivo*. Although it has been assumed that these are connected, some studies have refuted this idea. It has been suggested that human RDGBα (Nir2, also known as PITPNM1) is required to maintain DAG levels at the Golgi by modulating the CDP-choline pathway (Litvak et al., 2005) although a more recent study in human cells has shown that Nir2 functions at the plasma membrane (Chang et al., 2013). A previous study of the PITP domain of *Drosophila* RDGB has also suggested a lack of correlation between PtdIns binding and transfer *in vitro* and its function *in vivo* (Milligan et al., 1997). Thus, the biochemical function of the PITP domain and its role in supporting PLC-mediated signalling remains disputed.

**Fig. 1.**
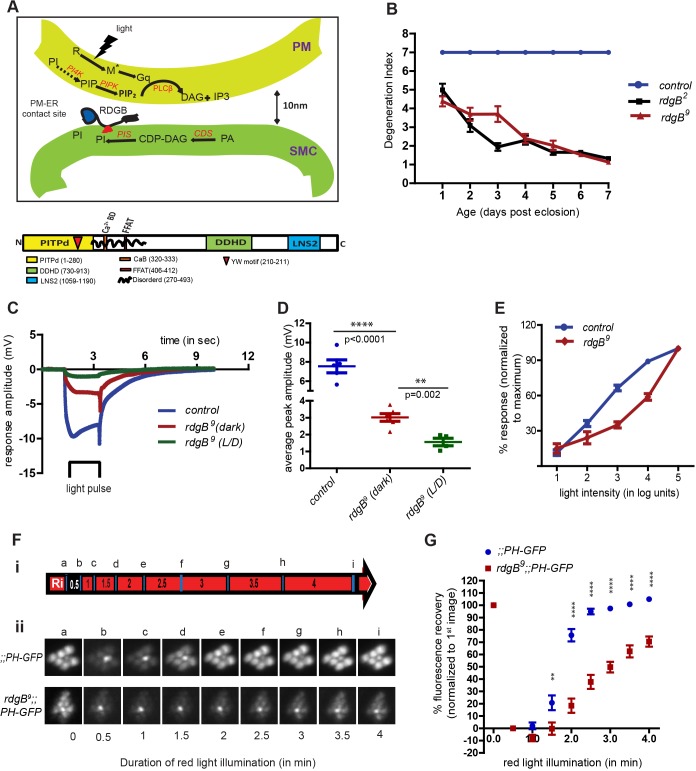
**RDGB is required to support multiple aspects of photoreceptor function.** (A) Schematic diagram representing the sensory PLC signalling pathway and the associated PtdIns(4,5)*P*_2_ resynthesis pathway organised between the plasma membrane (PM) and the adjacent submicrovillar cisternae (SMC). R, Rhodopsin; M*, metarhodopsin; Gq, G-protein; lipids and proteins are in black, whereas the enzymes are indicated in red; solid arrows represent demonstrated conversions and dotted arrows represent predicted conversions. The lower panel shows the domain architecture of RDGB (1259 amino acids). (B) Timecourse of retinal degeneration in *rdgB^2^* (protein null) and *rdgB^9^* (hypomorph) flies. The degeneration index represents the mean±s.e.m. number of intact photoreceptors per ommatidium (*n*=50 ommatidia counted from at least 5 different flies). (C) Representative ERG trace of 1-day-old flies of indicated genotype. The bar represents the time of light exposure. All the flies were reared in incubators with no illumination. *rdgB^9^* was shifted to a 12-h-light–12-h-dark cycle (L/D) cycle for 1 day post eclosion. (D) Quantification of the light response. Average of the peak amplitude of ERG recorded from 1-day-old flies. Control flies were reared in the dark. For *rdgB^9^*, recordings were performed for both dark-reared flies (dark) and for flies exposed to 12-h-light–12-h-dark cycle (L/D) post eclosion. Each data point represents an individual fly tested. Error bars represent s.e.m. *P*-values were calculated using a Student's *t*-test. (E) Intensity response function of 1-day-old *rdgB^9^* and wild-type flies. The mean±s.e.m. response is depicted over a range of light intensities separated by 1 log unit each. The *y*-axis shows the average response at any given intensity of light, as a percentage of the response recorded at maximum light intensity. Each data point represents *n*=7 for control and *n*=10 for *rdgB^9^*. (Fi) Schematic of the protocol used for live pseudopupil imaging. ‘R_i_’ represents the initial exposure to red light for 6 min. The red colour represents exposure to red light and black represents dark. Each blue bar represents a 90-ms flash of blue light used for image acquisition. Numbers within each box depict the time duration in minutes. Lowercase letters refer to individual images acquired at respective time points. (Fii) Representative fluorescent pseudopupil images acquired at the indicated time points (as shown in the schematic) from wild-type and *rdgB^9^* flies. (G) Recovery kinetics of fluorescent pseudopupil with time: the *y*-axis represents the mean±s.e.m. (*n*=4) fluorescence intensity of each pseudopupil image expressed as percentage of the intensity of first image acquired. *P*-values were calculated using two-way ANOVA with Bonferroni post test corrections.

Sensory transduction in *Drosophila* photoreceptors has been a useful model for analysis of PLC signalling *in vivo* (Hardie and Raghu, 2001). In these cells, photon absorption by the GPCR rhodopsin is transduced into electrical activity by G-protein-coupled PLCβ-mediated PtdIns(4,5)*P*_2_ hydrolysis (Fig. 1A) (Raghu and Hardie, 2009; Raghu et al., 2012). Photoreceptors have high basal PLC activity and, during bright light illumination, PLCβ activity and consequent PtdIns(4,5)*P*_2_ hydrolysis are substantially enhanced (Hardie and Raghu, 2001). Thus, in order to maintain their ability to respond to continuous illumination, PtdIns(4,5)*P*_2_ needs to be resynthesised to match its consumption by PLCβ activity. To date, there is no report identifying the lipid phosphatase activity that acts on rhabdomeral PtdIns(4,5)*P*_2_ during PLC signalling. However, consistent with the requirement of PITPs in PLC signalling, *Drosophila* photoreceptors are enriched for the PITP-domain-containing protein RDGB (Vihtelic et al., 1993).

*rdgB* mutants exhibit defective light responses and light-dependent retinal degeneration (reviewed in Trivedi and Padinjat, 2007). However, the molecular basis for the requirement of RDGB in these cells remains unresolved; a previous study [using Kir channels as biosensors to report PtdIns(4,5)*P*_2_ levels] reported delayed recovery of PtdIns(4,5)*P*_2_ levels in *rdgB* mutant photoreceptors following light stimulation (Hardie et al., 2001). It has also been reported that the PITP domain of *rdgB* is sufficient to rescue mutant phenotypes, yet the same study reported that a mutant version of this PITP domain (T59E) was able to transfer PtdIns *in vitro* but was unable to rescue function (Milligan et al., 1997). Here, we have carried out a comprehensive analysis of the lipid binding and transfer properties of the PITP domain of RDGB (RDGB-PITPd) *in vitro* and compared these with its ability to rescue *rdgB* mutant phenotypes *in vivo*. We find that RDGB-PITPd has distinctive lipid binding and transfer properties *in vitro* and regulates levels of two key lipids, namely PtdOH and PtdIns(4,5)*P*_2_, during PLC signalling.

## RESULTS

### RDGB is required to support normal phototransduction

**Fig. 2.**
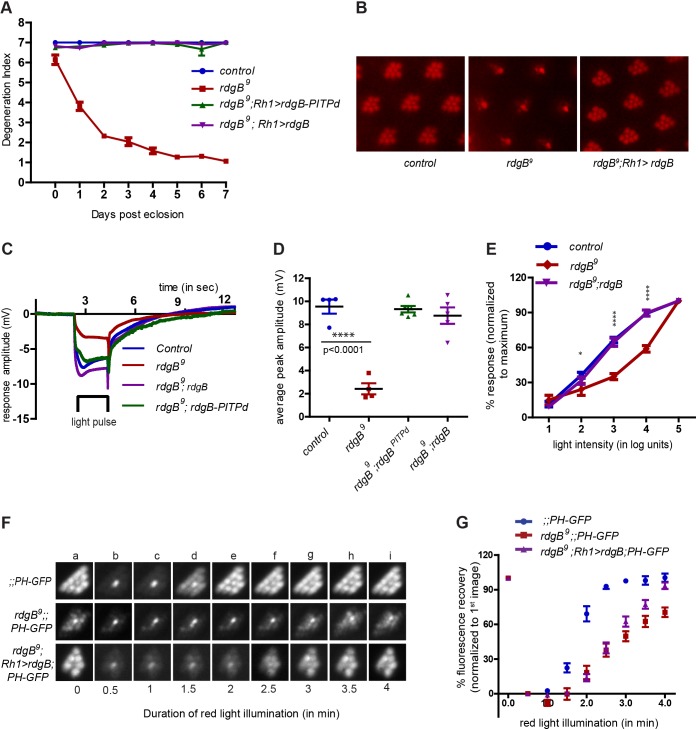
**Expression of RDGB rescues all the phenotypes of *rdgB^9^*.** (A) Degeneration index of the indicated genotypes over a period of 7 days. All the conditions are as described for Fig. 1. Error bars represent s.e.m. (B) Representative optical neutralisation images of 3-day-old flies of the indicated genotype. (C) Representative ERG trace from 1-day-old flies of indicated genotypes. The bar represents the time of light exposure. (D) Quantification of the light response. The mean±s.e.m. peak amplitude of ERG response of 1-day-old flies of indicated genotype is shown. Each data point represents an individual fly tested. *P*-values were calculated using a Student’s *t*-test. (E) Intensity response function of 1-day-old flies of the indicated genotype. The mean±s.e.m. response is depicted over a range of light intensities separated by 1 log unit each. The *y*-axis shows the average response at any given intensity of light, as a percentage of the response recorded at maximum light intensity. *n*=5 for all genotypes. (F) Representative fluorescent pseudopupil images acquired at different time points during pseudopupil imaging of the indicated genotypes. (G) Recovery kinetics of fluorescent pseudopupil with time for indicated genotypes. Data points represent mean±s.e.m. (n=4 for *rdgB^9^*, *n*=5 for other genotypes)*. P*-values were calculated using two-way ANOVA with Bonferroni post test corrections.

Although *rdgB* mutants show both light-dependent retinal degeneration and defective electrical responses to light (Harris and Stark, 1977), the relationship between these two phenotypes is unclear, given that structural abnormalities in a degenerating photoreceptor are likely to result in an abnormal light response. This has raised questions about the independent role of *rdgB*, if any, in supporting phototransduction. We used optical imaging to visualise rhabdomere integrity in intact retinae (Franceschini and Kirschfeld, 1971). Two alleles of *rdgB*, namely *rdgB^2^* (a protein null allele) (Vihtelic et al., 1991) and *rdgB^9^* (a severe hypomorph), were tested. As previously reported, peripheral photoreceptors underwent complete loss of rhabdomeres by day 5, whereas the central UV-sensitive photoreceptor was spared. The timecourse of degeneration was similar in both *rdgB^2^* and *rdgB^9^* (Fig. 1B). This degeneration could be rescued by expression of either the full-length wild-type *rdgB* transgene or the PITP domain of *rdgB* (*rdgB-PITPd*) (Fig. 2A,B). These findings were confirmed by examination of photoreceptor ultrastructure in sections of fixed samples (supplementary material Fig. S1A,B). We studied the response of photoreceptors to light using electrical recordings from the eye (by electroretinogram, ERG). There have been several reports (Harris and Stark, 1977; Rubinstein et al., 1989) demonstrating negligible electrical responses to light in *rdgB* mutants. However, most studies to date have analysed flies older than 2–3 days, by which time substantial retinal degeneration has set in (Fig. 1B). Therefore we tested light responses of young flies reared in the dark prior to the onset of any detectable retinal degeneration (age ≤20 h posteclosion). Under these conditions, *rdgB^9^* showed a reduced ERG amplitude (3±0.5 mV compared to 9±0.5 mV in wild type) (Fig. 1C). When exposed to a light–dark cycle for 1 day posteclosion, light responses from *rdgB^9^* were further reduced (Fig. 1C,D); under equivalent conditions wild-type responses were unaffected. This reduction in ERG amplitude could be rescued by either the full-length wild-type *rdgB* transgene or the PITP domain of *rdgB* alone (Fig. 2C,D). We also studied the sensitivity of *rdgB^9^* photoreceptors to light by recording the response to increasing intensities of light stimulus. Compared to wild-type controls of matched eye colour, *rdgB^9^* photoreceptors showed a substantially reduced sensitivity to light (Fig. 1E) and this could be rescued by a wild-type *rdgB* transgene (Fig. 2E). These findings demonstrate the existence of phototransduction defects in *rdgB^9^* prior to the onset of any detectable structural abnormality.

### RDGB function is required to support light-activated PtdIns(4,5)*P*_2_ turnover

The protein encoded by *rdgB* has a PITP domain (Fig. 1A) suggesting that during phototransduction RDGB might regulate PtdIns metabolism including PtdIns(4,5)*P*_2_ resynthesis at the plasma membrane. To monitor changes in plasma membrane PtdIns(4,5)*P*_2_ levels during PLC stimulation we used a well-established probe (Várnai and Balla, 1998), the PH domain of PLCδ (PH-PLCδ) fused to GFP (PH-PLCδ–GFP) (Chakrabarti et al., 2015; Sengupta et al., 2013). In *Drosophila* photoreceptors, this probe is uniformly distributed on the plasma membrane (supplementary material Fig. S2F). Given that the phototransduction machinery is limited to the apical plasma membrane (rhabdomeres) and we were interested in monitoring changes in PtdIns(4,5)*P*_2_ levels at the rhabdomere, we exploited the technique of pseudopupil imaging, which reports molecules present in the rhabdomere (Franceschini and Kirschfeld, 1971) (see schematic of protocol used in Fig. 1Fi). Under resting conditions, the probe binds to PtdIns(4,5)*P*_2_ and hence localises to the microvillar plasma membrane giving rise to a fluorescent pseudopupil (Fig. 1Fii, panel a). Following a flash of blue light that triggers PLC activity, microvillar PtdIns(4,5)*P*_2_ is depleted and the probe is displaced from the plasma membrane and diffuses out of the microvilli, resulting in loss of the fluorescent pseudopupil (Fig. 1Fii, panel b). (The central photoreceptor, R7 is not stimulated by blue light and hence retains the probe.) In the dark, presumably following PtdIns(4,5)*P*_2_ resynthesis, the probe relocalises to the microvillar plasma membrane resulting in the recovery of the fluorescent pseudopupil. Increasing durations of red light illumination (that converts metarhodopsin into rhodopsin, thus terminating PLC activity) following each image acquisition results in progressively faster recovery of fluorescence (Fig. 1Fii, panels c–i). In 1-day-old *rdgB^9^* flies, the fluorescent pseudopupil is diffuse and lower in intensity to begin with [despite western blotting showing equivalent level of probe expression (supplementary material Fig. S1C)] and following stimulation with blue light recovers with a much slower timecourse (Fig. 1G). These observations suggest that RDGB is required both to maintain basal PtdIns(4,5)*P*_2_ levels at the micovillar plasma membrane and to support PtdIns(4,5)*P*_2_ resynthesis following light-induced PLC activation.

We tested the ability of RDGB to rescue the PtdIns(4,5)*P*_2_ resynthesis defect of *rdgB^9^* photoreceptors. Expression of RDGB in *rdgB^9^* photoreceptors was able to rescue both the basal pseudopupil fluorescence (supplementary material Fig. S1D,E) as well as allowing it to recover back to 100% albeit with delayed kinetics (Fig. 2F,G); this delay was presumably due to a lower level of expression of RDGB from the transgenic construct (supplementary material Fig. S2A).

### The PITP domain of RDGB binds and transfers both PtdIns and PtdOH *in vitro*

**Fig. 3.**
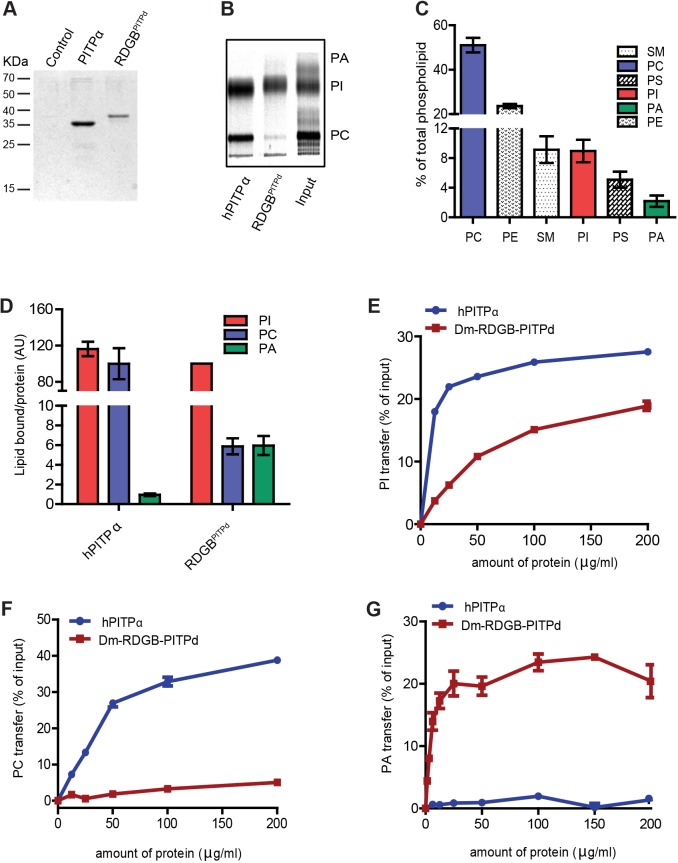
**Characterisation of the lipid-binding properties of the PITP domain of RDGB.** (A) Image of an SDS-PAGE gel showing captured PITPs after incubation with ^14^C-labelled permeabilised HL60 cells. (B) Thin-layer chromatography (TLC) showing the lipids bound to the captured proteins. His-tagged RDGB and hPITPα (120 μg) were incubated for 20 min with pre-permeabilised HL60 cells (∼10^7^ cells) pre-labelled with [^14^C]acetate for 48 h. Recombinant proteins with bound lipids were captured using nickel beads after separation of cells. (C) The lipid composition of HL60 cells showing the relative amounts of different phospholipids. Error bars represent s.d. (*n*=5). (D) The relative binding affinity of hPITPα and RDGB for PtdIns (PI), PtdCho (PC) and PtdOH (PA). The amount of lipid bound was normalised to the amount of recombinant protein recovered. For normalisation, PtdIns bound by wild-type RDGB-PITPd was set at 100%. Error bars represent s.d. (*n*=3). (E–G) Lipid transfer activity of hPITPα and RDGB for PtdIns, PtdCho and PtdOH. The *y*-axis in each represents amount of lipid transferred as a percentage of the total input count. Error bars represent s.d. (*n*=2, experiments repeated three times with *n*=2 each). SM, sphingomyelin; PC, PtdCho; PS, PtdSer; PI, PtdIns; PA, PtdOH; PE, PtdEtn.

We expressed and purified RDGB-PITPd from *E. coli* and examined its lipid binding and transfer activities *in vitro* in comparison to the well-characterised human PITPα (hPITPα, also known as PITPNA) protein. Unlike hPITPα, which can bind either a molecule of phosphatidylcholine (PtdCho) or PtdIns, RDGB-PITPd showed limited PtdCho binding (Fig. 3A,B). Further, as recently reported for human RDGBβ (Garner et al., 2012), we found that RDGB-PITPd was able to bind PtdOH (Fig. 3D). These binding studies were conducted in permeabilised HL60 cells, where the recombinant proteins were exposed to the cellular phospholipids present at their physiological levels. PtdOH represents a very small fraction (<1–2%) of the total phospholipids (Fig. 3C). Nonetheless, RDGB-PITPd bound a substantial amount of PtdOH. PtdCho is also bound by RDGB-PITPd to a similar level, but considering that in cells PtdCho represents nearly 50% of the total lipids, the affinity for PtdCho is likely to be significantly lower than that for PtdOH. PtdIns, which represents ∼5–8% of the total cellular lipids, was the dominant lipid bound to RDGB-PITPd (Fig. 3D). In contrast, hPITPα bound PtdIns and PtdCho equivalently, and insignificant binding to PtdOH was observed (Fig. 3D). When the binding of each lipid to the PITP was normalised to the total amount of lipid available, it was clear that both hPITPα and RDGB-PITPd had a higher affinity for PtdIns compared to PtdOH or PtdCho (supplementary material Fig. S2E). The most important difference between the class I PITPα and class II RDGB-PITPd is that the class I protein has a preference for binding PtdCho whereas RDGB-PITPd has a strong preference for PtdOH (supplementary material Fig. S2E).

We also performed *in vitro* transfer assays and compared the activity of RDGB-PITPd with that of hPITPα (Ségui et al., 2002; Tilley et al., 2004). Under equivalent conditions, both RDGB-PITPd and hPITPα showed PtdIns transfer activity (Fig. 3E); compared to hPITPα, RDGB-PITPd was less active. Whereas hPITPα showed substantial PtdCho transfer activity, RDGB-PITPd showed very little transfer activity towards PtdCho (Fig. 3F). In sharp contrast, RDGB-PITPd showed substantial PtdOH transfer activity, whereas hPITPα was unable to transfer PtdOH (Fig. 3G). Taken together, these data show that, *in vitro*, RDGB-PITPd can bind and transfer both PtdIns and PtdOH.

### Molecular basis of PtdIns binding and transfer by the PITP domain *in vitro*

**Fig. 4.**
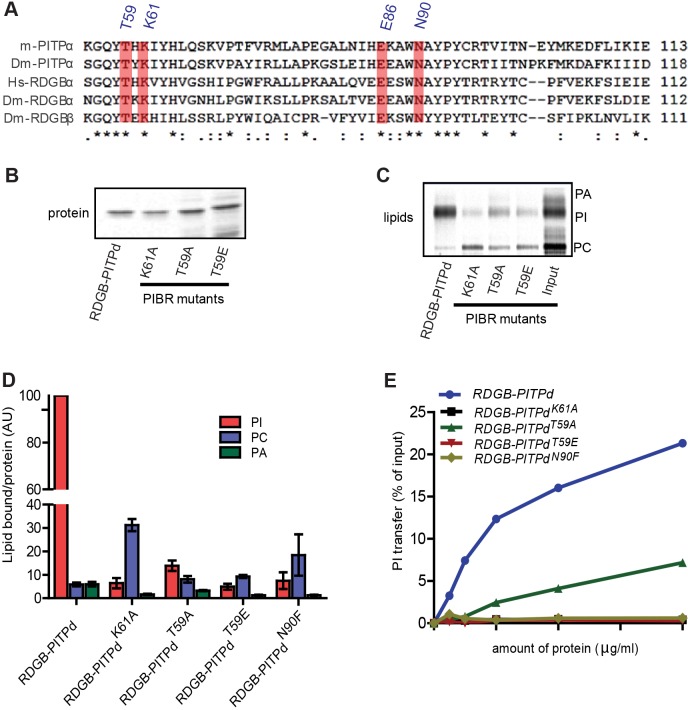
**Lipid-binding and transfer properties of PIBR mutant proteins.** (A) Alignment of the indicated PITPs showing conservation of residues within the PITP domain that coordinate with the inositol head group of PtdIns. Highlighted residues (T59, K61, E86 and N90) are conserved across all sequences *, identical residues; :, conservative substitution with strongly similar properties; ., conservative substitution with weakly similar properties. (B) Image of an SDS-PAGE gel showing captured RDGB proteins after incubation with the permeabilised HL60 cells. (C) Thin-layer chromatography (TLC) showing the lipids bound to the captured proteins. (D) Quantification of the lipids bound to wild-type and mutant RDGB-PITPd proteins expressed as a ratio of lipid bound [PtdCho (PC), PtdIns (PI), PtdOH (PA)] to protein recovered. Error bars are s.d. (*n*=5). (E) Comparison of the PtdIns transfer activity of wild-type RDGB-PITPd and PIBR mutant proteins with different concentrations of recombinant protein used. Error bars represent s.d. (*n*=2).

A conserved property of the PITP domain (across all classes) is the ability to bind and transfer PtdIns *in vitro*. The structural basis of PtdIns binding has previously been investigated for hPITPα (Tilley et al., 2004). The crystal structure of PITPα reveals the identity of those amino acid residues in this protein that directly co-ordinate with the inositol ring of PtdIns and appear to be essential for both *in vitro* and *in vivo* function, hereafter called PtdIns-binding residues (PIBRs) (Tilley et al., 2004). These PIBRs are conserved in nearly every metazoan PITP domain (that has been sequenced) regardless of the class of PITPs to which the parent protein belongs to, presumably reflecting an evolutionarily conserved role in the function of this domain (Tilley et al., 2004) (Fig. 4A). We studied the requirement of three of these PIBRs, namely T59, K61 and N90 (mouse PITPα numbering used throughout), for the binding of PtdIns to RDGB-PITPd. The single point mutants K61A, N90F and, in the case of T59, two versions T59A and T59E were generated in RDGB-PITPd, then expressed in bacteria and purified (Fig. 4B), and their biochemical properties were studied *in vitro*. We found that all the four mutant proteins (RDGB-PITPd^T59A^, RDGB-PITPd^T59E^, RDGB-PITPd^K61A^ and RDGB-PITPd^N90F^) showed a dramatically diminished ability to bind PtdIns compared to wild-type RDGB-PITPd (Fig. 4C,D). The T59A, T59E and N90F mutants did not show altered PtdCho binding, whereas in the case of K61A, the lowered PtdIns binding was associated with an increase in PtdCho binding. Notably T59A bound more PtdIns than T59E (Fig. 4C,D). In all mutant versions of RDGB-PITPd, PtdOH binding was reduced (Fig. 4C,D).

We also studied the transfer activity of each PIBR mutant using an *in vitro* PtdIns transfer assay and found that, with the exception of T59A, all other mutant versions of RDGB-PITPd showed essentially no PtdIns transfer activity (Fig. 4E). T59A retained some residual PtdIns transfer activity, although this was substantially reduced compared to the wild-type protein. These results differ from those reported before for the PITP domain of RDGB. RDGB-PITPd^T59A^ has previously been reported to be null for PtdIns transfer whereas RDGB-PITPd^T59E^ was reported to have activity comparable to wild type (Milligan et al., 1997). Our results presented here are in accordance with what has been reported for PITPα-T59A (Tilley et al., 2004). The PtdOH transfer activity of the mutant proteins was also analysed and was largely unaffected at saturating concentrations of protein (supplementary material Fig. S2B); at very low concentrations, PtdOH transfer by the mutants was marginally reduced. We noted a discrepancy between the lipid binding and transfer activities described here. The biochemical assays for monitoring lipid binding and transfer were undertaken in different membrane environments and therefore represent different properties of the protein. Lipid binding was carried out in permeabilised cells, whereas lipid transfer monitored the movement of radiolabelled lipid from a donor membrane (microsomes or liposomes) to an acceptor membrane (liposomes or mitochondria). Thus, PtdOH binding is modest for RDGB-PITPd, but PtdOH transfer is substantial (Fig. 3). In the PIBR mutants, PtdOH binding is highly reduced although PtdOH transfer is only marginally affected. A small amount of PtdCho is bound by RDGB-PITPd but is unable to transfer PtdCho. This discrepancy has been observed previously for the WW/AA mutants of PITPα (Tilley et al., 2004) and is also evident for the analogous YW/AA mutants described here (see Fig. 8A–F).

### PtdIns-binding residues are essential to support RDGB function *in vivo*

**Fig. 5.**
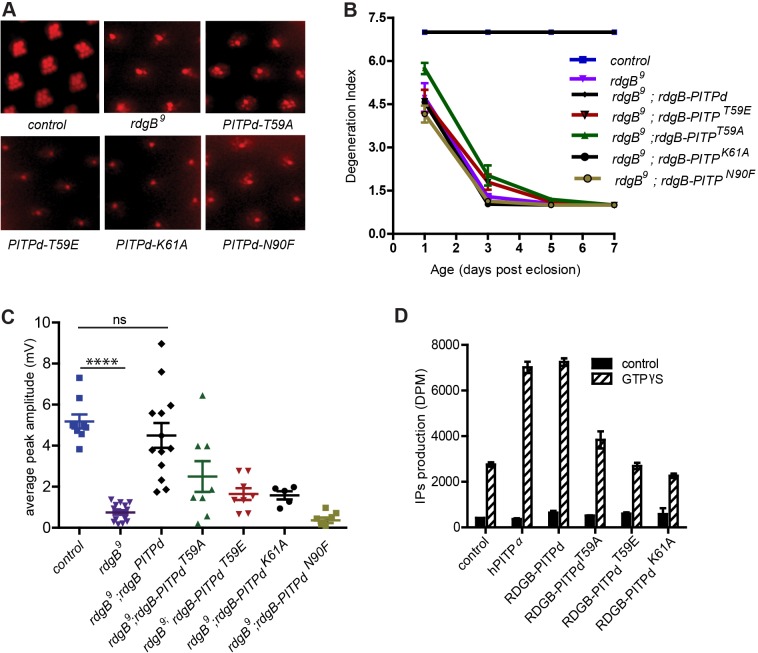
**PtdIns-binding residue mutants are unable to rescue *rdgB^9^.*** (A) Representative images of optical neutralisation performed on 3-day-old flies of indicated genotype. (B) Degeneration index of *rdgB^9^* compared to *rdgB^9^* photoreceptors reconstituted with the indicated PIBR mutants. Data points represent mean±s.e.m. (*n*=50 ommatidia counted from at least five different flies). (C) Mean±s.e.m. peak amplitude of ERG responses from 1-day-old *rdgB^9^* flies compared to *rdgB^9^* flies reconstituted with the indicated PIBR mutant. The results are shown as data points for the respective genotype. *P*-values were calculated with a Student’s *t*-test. (D) Ability of hPITPα, RDGB-PITPd and PIBR mutant proteins to support G-protein-coupled PLC activity in permeabilised cells stimulated with GTPγS. Error bars represent s.d. (*n*=3).

We tested the requirement of PIBRs in supporting the function of RDGB *in vivo*. Transgenic flies were generated that allowed the expression of either wild-type RDGB-PITPd or each of the PIBR mutants of RDGB-PITPd in photoreceptors. Transgenic lines that showed equivalent level of protein expression (supplementary material Fig. S2C) were used in the following experiments. We studied two phenotypes of *rdgB*, namely retinal degeneration and the electrical response to light. As previously indicated (Milligan et al., 1997) and in this study (Fig. 2A,D), RDGB-PITPd was able to rescue the retinal degeneration phenotype of *rdgB^9^*. By contrast, none of the mutant versions (RDGB-PITPd^T59A^, RDGB-PITPd^T59E^, RDGB-PITPd^K61A^ and RDGB-PITPd^N90F^) were able to rescue retinal degeneration in *rdgB^9^* (Fig. 5A,B). In the case of RDGB-PITPd^T59A^, the rate of retinal degeneration was somewhat slower compared to *rdgB^9^*.

We also tested the requirement of PIBRs to support a normal electrical response to light. ERGs were recorded from *rdgB^9^* reconstituted with either wild-type RDGB-PITPd or a mutant version of each of the PIBRs. We found that although RDGB-PITPd was able to rescue the light response of *rdgB^9^* to near wild-type levels, none of the PIBR mutant versions were able to do so (Fig. 5C). Unlike the case with the retinal degeneration phenotype, RDGB-PITPd^T59A^ was not able to improve the light response in *rdgB^9^*. These results suggest that binding of PtdIns to the RDGB-PITPd is essential to support a normal response to light in photoreceptors.

In mammalian cells, PITPα has been shown to be required to support G-protein-coupled PtdIns(4,5)*P*_2_ turnover in permeabilised HL60 cells (Thomas et al., 1993). We studied this with respect to the function of RDGB-PITPd. Using the permeabilised cell assay, we found that RDGB-PITPd was able to stimulate the production of inositol 1,4,5-trisphosphate (IP_3_) in response to GTPγS stimulation just as well as PITPα (Fig. 5D). However, in this assay, RDGB-PITPd^T59E^ and RDGB-PITPd^K61A^ were not able to support the production of IP_3_ (Fig. 5D). RDGB-PITPd^T59A^ retained partial activity, in keeping with the PtdIns transfer activity observed for this mutant (Fig. 4E). These observations suggest that, similar to PITPα (Tilley et al., 2004), the PtdIns-binding activity of RDGB-PITPd is required to support G-protein-stimulated inositol lipid turnover.

**Fig. 6.**
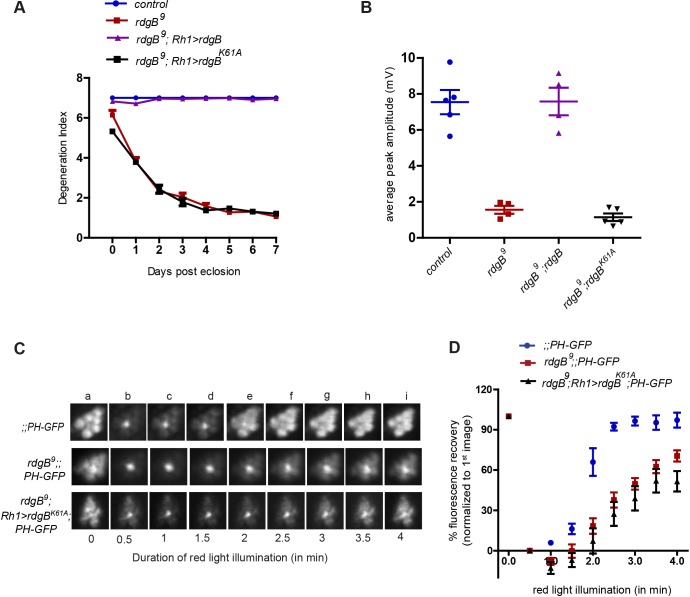
***rdgB^K61A^* is unable to rescue *rdgB^9^*.** (A) Degeneration index (mean±s.e.m.) of the indicated genotypes over a period of 7 days (*n*=50 ommatidia counted from at least five different flies). (B) Mean±s.e.m. peak amplitude of the ERG response of 1-day-old flies of the indicated genotype. The results are shown as data points. (C) Representative images acquired at different time points during fluorescent pseudopupil imaging of the indicated genotypes. (D) Recovery kinetics of fluorescent pseudopupil with time for indicated genotypes. Data points represent mean±s.e.m (*n*=4). *P*-values were calculated using two-way ANOVA with Bonferroni post test corrections.

Given that RDGB is a large protein (160 kDa) compared to its PITP domain alone (35 kDa), we also generated one of the PIBR mutant forms (K61A) in the context of full-length *rdgB* (*rdgB^K61A^*) and tested its ability to rescue *rdgB^9^* phenotypes. *rdgB^K61A^* showed similar behaviour to *rdgB-PITPd^K61A^* and could not rescue the retinal degeneration and ERG phenotypes of *rdgB^9^* (Fig. 6A,B). We also tested the ability of *rdgB^K61A^* to rescue the PtdIns(4,5)*P*_2_ resynthesis defect of *rdgB^9^*. Under conditions where RDGB was able to rescue the PtdIns(4,5)*P*_2_ resynthesis defect of *rdgB^9^*, reconstitution of *rdgB^9^* with *rdgB^K61A^* was unable to reverse the delayed kinetics of PtdIns(4,5)*P*_2_ turnover (Fig. 6C,D). Collectively these results imply that PtdIns binding is an essential requirement of for the *in vivo* function of RDGB.

### A *Drosophila* class I PITP cannot substitute for RDGB function *in vivo*

**Fig. 7.**
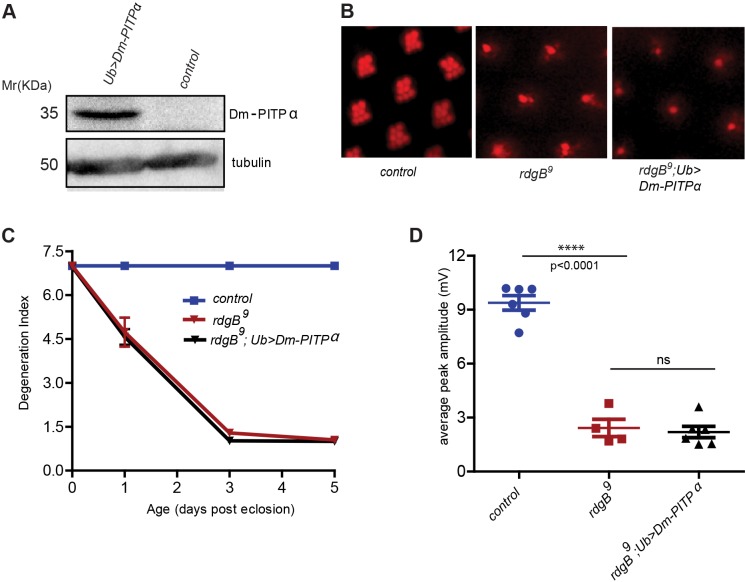
**A *Drosophila* class I PITP is unable to rescue the mutant phenotypes of *rdgB^9^.*** (A) Western blot of head extracts made from wild-type and *Dm-PITPα*-overexpressing flies, showing expression of PITPα. The blot was re-probed with anti-tubulin antibody as a loading control. (B) Representative images of optical neutralisation performed on 3-day-old flies of the indicated genotype. (C) Degeneration index (mean±s.e.m.; *n*=50 ommatidia counted from at least five different flies) of mentioned genotypes over a period of 5 days. *Dm-PITPα* was overexpressed using the *Ub* promoter. (D) Mean±s.e.m. peak amplitude of the ERG response of 1-day-old flies of the indicated genotype. The results are shown as data points. The *P*-value was calculated using a Student's *t*-test.

Class I and class II PITPs show distinct lipid binding and transfer properties (Fig. 3), but a conserved feature is the ability to bind and transfer PtdIns *in vitro*. Given that we found that PtdIns binding was essential for RDGB function *in vivo*, we hypothesised that if this biochemical property was the only determinant of RDGB function, then expression of another PITP should rescue *rdgB* phenotypes. Remarkably, it has been previously reported that mammalian PITPα or a PITPα-RDGB chimera is unable to restore the light response or rescue retinal degeneration when expressed in *rdgB^2^* flies (Milligan et al., 1997). However, it was not clear whether the lack of rescue by mammalian PITPα in that study was due to evolutionary diversification of PITPα between *Drosophila* and mammals. To resolve this issue we tested the ability of a *Drosophila* class I PITP to rescue the phenotypes of *rdgB^9^*. The *Drosophila* genome encodes only one member of class I PITP, Dm-PITPα (i.e. *vib*, CG5269) (Gatt and Glover, 2006; Giansanti et al., 2006). Endogenous Dm-PITPα is expressed in adult heads at very low levels, so we overexpressed it using the ubiquitin promoter (Fig. 7A). Under these conditions, Dm-PITPα was unable to rescue retinal degeneration and the light response of *rdgB^9^* (Fig. 7B–D). This observation strongly suggests that there are likely to be other biochemical properties in addition to PtdIns binding and transfer that might be unique to RDGB-PITPd function.

### An RDGB mutant that binds PtdIns but fails to transfer *in vitro* cannot support function *in vivo*

**Fig. 8.**
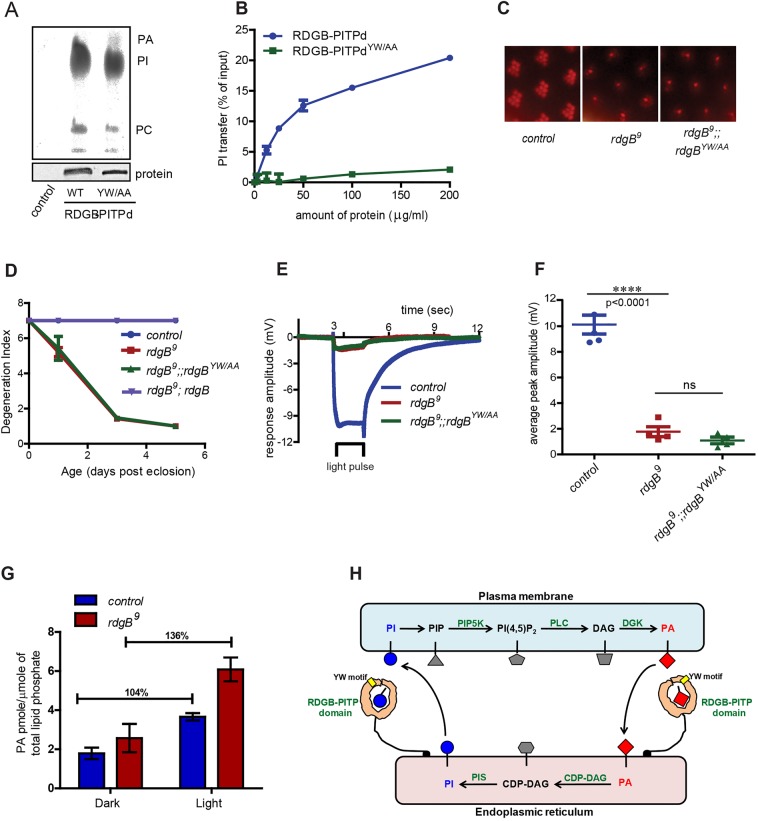
**Membrane docking is a prerequisite for the *in vivo* function of RDGB.** (A) Thin-layer chromatography (TLC) showing different lipids bound to the mentioned recombinant proteins after incubation with prelabelled HL-60 cells (upper panel). The lower panel shows western blot of a sample of the captured proteins. WT, wild type. (B) Comparison of the PtdIns transfer activity of wild-type RDGB-PITPd and RDGB-PITPd^YW/AA^ with different concentrations of recombinant protein. Error bars represent s.d. (*n*=2). (C) Representative images of optical neutralisation performed on 3-day-old flies of mentioned genotype. (D) Degeneration index of the indicated genotypes over a period of 5 days. Error bars represent s.e.m. (*n*=50 ommatidia counted from at least five different flies). (E) Representative ERG trace from 1-day-old flies of the indicated genotypes. The bar represents the time of light exposure. (F) Mean±s.e.m. peak amplitude of ERG response of 1-day-old flies of indicated genotypes. The results are shown as data points. *P*-values were calculated using a Student's *t*-test; ns, not significant. (G) Measurement of total PtdOH levels in heads of wild-type and *rdgB^9^* flies reared in dark and after 1 min of bright light stimulation. PtdOH level was normalised to the lipid phosphate content of sample. Values represent mean±s.e.m. (H) Cartoon depicting the role of RDGB in transporting PtdIns (PI) and PtdOH (PA) across the plasma membrane and ER during PLC signalling. The lipid intermediates of the PtdIns(4,5)*P*_2_ cycle are marked tethered to the membrane at which they are localised. Enzymes are marked in green. The RDGB protein with its lipid-bound PITP domain is shown; the YW motif essential for the membrane docking step is marked. Arrows indicate the presumed direction of lipid transfer.

To date there is no agreement on the contribution of PtdIns binding and transfer activity for the *in vivo* function of PITPs. Although PIBR mutants do not transfer PtdIns *in vitro*, presumably this reflects their inability to bind PtdIns. Thus, a mutant that retains PtdIns binding but cannot perform PtdIns transfer activity *in vitro* could help address the importance of transfer activity *in vivo*. It has been proposed that PITP function requires the essential step of membrane docking, and two residues present within the PITP domain, namely W203 and W204 (mice PITPα numbering), have been implicated in this event (Schouten et al., 2002; Shadan et al., 2008; Tilley et al., 2004; van Tiel et al., 2002). Sequence analysis showed that this ‘tryptophan motif’ is conserved across all classes of PITPs with the minor difference that all class II PITPs have a tyrosine in place of the first tryptophan residue of the motif. In case of RDGB, this YW motif is present at position 210 and 211. We mutated both these residues to alanine (YW/AA) and tested the ability of the recombinant protein to bind and transfer lipid *in vitro*. We found that RDGB-PITPd^YW/AA^ was able to bind PtdIns at levels comparable to RDGB-PITPd (Fig. 8A). However, the PtdIns transfer activity of RDGB-PITPd^YW/AA^ was profoundly reduced (Fig. 8B). These *in vitro* properties of the YW/AA mutant are similar to those previously reported for the WW/AA mutants both in case of PITPα and for PITPβ (also known as PITPNB) (Shadan et al., 2008; Tilley et al., 2004). Thus, RDGB-PITPd^YW/AA^ represents a protein that can bind PtdIns but not transfer it *in vitro*.

To test the importance of PtdIns transfer *in vivo* we generated transgenic flies and tested the ability of RDGB^YW/AA^ to rescue *rdgB^9^*. We found that RDGB^YW/AA^ was neither able to rescue the light response nor was able to suppress retinal degeneration in *rdgB^9^* (Fig. 8C–F). These observations demonstrate that a RDGB^YW/AA^ has an intrinsic ability to bind PtdIns but is unable to transfer *in vitro* and cannot rescue *in vivo* function.

### RDGB function is required to regulate PtdOH levels during phototransduction

Given that Dm-PITPα, which can also bind and transfer PtdIns, was unable to rescue *rdgB^9^*, we hypothesised that the additional and unique biochemical activity of RDGB-PITPd that we found, namely PtdOH binding and transfer, might also be required to support *in vivo* function. If PtdOH binding and transfer activity of RDGB were important *in vivo*, levels of PtdOH and its immediate precursor DAG might be altered in *rdgB^9^* photoreceptors, especially in response to enhanced PLC stimulation. To test this we used high-resolution lipid mass-spectrometry to measure PtdOH and DAG levels from head extracts. In dark-adapted photoreceptors, PtdOH levels were almost equal between wild-type and *rdgB^9^* (Fig. 8G, also see supplementary materials Fig. S3). Following illumination with a brief (1 min) flash of bright light, PtdOH and DAG levels rose in controls, reflecting the production of these lipids by the sequential action of PLC and DGK (supplementary material Fig. S4). However the light-induced elevation in PtdOH levels seen in *rdgB^9^* was substantially greater than in wild type (Fig. 8G). These observations suggest that RDGB function is required to regulate the turnover of PtdOH generated during phototransduction.

## DISCUSSION

To maintain the sensitivity of receptor-activated PLC signalling cascades, PLC must have adequate levels of its substrate. PtdIns(4,5)*P*_2_, the substrate of PLC, is a low abundance lipid (there are ∼4000 PtdIns(4,5)*P*_2_ molecules/μm^2^ on the inner leaflet of the plasma membrane, i.e. 0.1–5% of inner bilayer lipids; Xu et al., 2003), and maintaining stable levels of PtdIns(4,5)*P*_2_ is especially challenging in cells that experience high rates of PLC activity; in the absence of tight regulation, membranes are likely to show depletion of PtdIns(4,5)*P*_2_. However, multiple studies monitoring PtdIns(4,5)*P*_2_ levels have found that the levels of this lipid are quite stable and any drops in its levels during ongoing agonist stimulation are transient (Balla et al., 2008; Hardie et al., 2001; Várnai and Balla, 1998; Willars et al., 1998). PtdIns(4,5)*P*_2_ hydrolysis triggers a sequence of five reactions collectively referred to as the PtdIns(4,5)*P*_2_ cycle (Fig. 1A) that culminates in the resynthesis of this lipid. In order to keep the PtdIns(4,5)*P*_2_ cycle running, two key lipid intermediates of the PtdIns(4,5)*P*_2_ cycle, PtdOH and PtdIns need to be exchanged between the plasma membrane and the ER. In the present study, we define a single protein RDGB whose PITP domain possesses the biochemical activity to bind and transfer both PtdOH and PtdIns *in vitro*. RDGB therefore represents a candidate that might be able to exchange lipid intermediates between the plasma membrane and ER and thus keep the PtdIns(4,5)*P*_2_ cycle running. In support of this idea, we find that *rdgB* mutants show delayed kinetics of PtdIns(4,5)*P*_2_ resynthesis following PLC activation and this defect is not rescued by the PtdIns-binding-deficient mutants, thus implying PtdIns binding to PITPd is crucial (Fig. 6C,D). A recent study has shown that in *Drosophila* photoreceptors, loss of PIP5K activity slows the kinetics of PtdIns(4,5)*P*_2_ resynthesis (Chakrabarti et al., 2015). Additionally, loss of *Drosophila* PIP5K results in a further reduction of the residual light response in *rdgB^9^*. These findings are consistent with a model where RDGB function is required to support the activity of PIP5K to generate PtdIns(4,5)*P*_2_ during *Drosophila* phototransduction. We found a clear correlation between the ability of RDGB to bind PtdIns *in vitro* and its ability to rescue *rdgB^9^*, strongly suggesting that PtdIns binding to RDGB-PITPd is relevant for function *in vivo*. Furthermore, our finding that the RDGB^YW/AA^ protein (which can bind PtdIns but cannot transfer it *in vitro*) was unable to rescue *rdgB^9^*, suggests that PtdIns transfer is very likely an important function of RDGB *in vivo*.

A second crucial step in the PtdIns(4,5)*P*_2_ cycle that requires lipid exchange is the transfer of PtdOH to the ER. To date there are few reports of proteins that can transfer PtdOH between membranes. In yeast, a complex of Ups1 and Mdm35 is able to transfer PtdOH across the mitochondrial space (Connerth et al., 2012) and Garner et al. has reported that PITPNC1, a related protein of the RDGB family, is able to bind and transfer PtdOH in mammalian cells (Garner et al., 2012). In this study, we found that RDGB-PITPd is able to bind and transfer PtdOH *in vitro*. Importantly, we found that following exposure to light (which activates PLC), *rdgB^9^*showed an enhanced elevation in PtdOH levels compared to wild-type flies, suggesting that the RDGB protein is required to facilitate PtdOH metabolism during phototransduction. Live imaging of PtdOH levels during phototransduction might help strengthen this idea but is presently limited by the absence of a suitable PtdOH probe. Hence, the specific mechanism by which RDGB participates in PtdOH metabolism is unclear. Our finding of a PtdOH transfer activity for RDGB-PITPd *in vitro* suggests that this protein could facilitate transfer of PtdOH from the plasma membrane to the ER where it would be metabolised by CDS into CDP-DAG. In *rdgB^9^* photoreceptors, the absence of this activity could result in a reduced availability of PtdOH for CDS to metabolise into CDP-DAG, thus explaining the enhanced elevation of PtdOH levels following illumination. In a previous study, *Drosophila* DGK (*rdgA*), which generates PtdOH from DAG has been shown to be localised to the submicrovillar cisternae (SMC) (Masai et al., 1997) raising the question of whether PtdOH transfer activity is required. RDGA is a large protein (∼1457 amino acids) and it is possible that it can act in trans across the 10-nm cytoplasmic gap between the microvillar plasma membrane and the SMC to access its substrate (DAG) produced at the plasma membrane. Such a mechanism by which an enzyme acts in trans to access a lipid substrate on another membrane has been shown for Sac1 at membrane contact sites in yeast (Stefan et al., 2011).

PITPα is a protein with PtdIns and PtdCho transfer activity. Although PtdIns binding and transfer activity is conserved between PITPα and RDGB-PITPd, PtdOH binding and transfer is unique to RDGB-PITPd. This biochemical difference might represent the mechanistic basis of the inability of Dm-PITPα to rescue *rdgB^9^* phenotypes *in vivo*. It has been reported that one of the mammalian RDGB homologs, Nir2, can rescue the phenotype of *rdgB^9^* (Chang et al., 1997). This observation is likely to be a reflection of the importance of PtdOH-binding activity of RDGB. Consistent with this model, the PITP domain of the mammalian ortholog of RDGB, Nir2 has been shown to bind PtdOH (Garner et al., 2012) and also transfer PtdOH *in vitro* (S.C., unpublished data). Furthermore, a distinct member of the mammalian class II family, RDGBβ, has been shown to bind and transfer PtdOH (Garner et al., 2012). Thus, class II PITPs represent PtdIns and PtdOH transfer proteins, in contrast to class I PITPs that are PtdIns and PtdCho transfer proteins.

What might be the role of RDGB in the subcellular context of photoreceptor function? Earlier work has shown that RDGB is localised to a small specialised sub-compartment of the ER, the SMC present at the base of the microvillar plasma membrane (Vihtelic et al., 1993). The SMC is separated from the plasma membrane by a cytoplasmic gap of ∼10 nm. Sequence analysis of RDGB does not reveal any possible transmembrane segments and we found that consistent with what has been reported for its mammalian ortholog Nir2 (Litvak et al., 2002), RDGB is a membrane-associated protein rather than a membrane integral protein (supplementary material Fig. S2D). Given that RDGB has an FFAT motif, it is likely that this motif is bound to VAP proteins [vesicle-associated membrane protein (VAMP)-associated protein] present on the surface of SMC thereby anchoring it to the SMC. Conceptually, the spatial organisation of the SMC and the microvillar plasma membrane represents an example of a plasma-membrane–ER contact site (Levine, 2004) at which exchange of material might occur. The known localisation of RDGB to this site and the observation that RDGB-PITPd binds both PtdIns and PtdOH raises the possibility that RDGB-PITPd might be a key requirement for these lipids to move across the plasma membrane and SMC (Fig. 8H). Such movement of PtdOH and PtdIns would be required to prevent rundown of the PtdIns(4,5)*P*_2_ cycle. Alternatively, in this subcellular setting, RDGB-PITPd might sample the plasma membrane and its binding to PtdOH and/or PtdIns might transmit information to the ER machinery to stimulate PtdIns synthesis following PtdIns(4,5)*P*_2_ hydrolysis. Given that the enzymes involved in supporting *Drosophila* phototransduction are present across two distinct compartments (i.e. the plasma membrane and SMC) it is likely that RDGB acts as a sensor communicating the status of PtdIns(4,5)*P*_2_ turnover at the microvillar plasma membrane to the ER through its ability to bind PtdIns and/or PtdOH. In summary, our study demonstrates that the PITP domain of RDGB is important to support PtdIns(4,5)*P*_2_ resynthesis as well as to regulate PtdOH levels at the photoreceptor plasma membrane during G-protein-coupled PLC activation. The design of a protein such as RDGB that can bind and transfer both PtdOH and PtdIns might represent an elegant solution to monitor and/or exchange two key metabolic intermediates required to keep the PtdIns(4,5)*P*_2_ cycle running.

## MATERIALS AND METHODS

### Fly stocks

Flies (*Drosophila melanogaster*) were grown on standard corn meal medium with 1.5% yeast and were reared at 25°C in incubators with no internal illumination. *Ub-vib*-expressing flies were obtained from David Glover, University of Cambridge, UK.

### Generation of point mutants and transgenic flies

PtdIns-binding point mutants as well as the YW/AA mutation were generated in *rdgB-PITPd* by site-directed mutagenesis. The entire open reading frame was sequence-verified post-mutagenesis. Transgenic flies were generated (Rubin and Spradling, 1982) and lines showing equivalent expression levels were used in experiments.

### Protein expression for biochemical assays

The *Drosophila* RDGB-PITPd cDNA coding for the N-terminal PITP domain (1–281 amino acids of GI no. 15291155) was cloned into pRSET-C introducing an N-terminal His tag. Proteins were expressed and purified as described previously (Garner et al., 2012).

### Optical neutralisation

Optical neutralisation was performed as described previously (Franceschini and Kirschfeld, 1971). A total of 50 ommatidia were scored across five different flies. For all experiments flies were reared in the dark and transferred to 12-h-light–12-h-dark regime post eclosion.

### Electrophysiology

External electrical recordings were performed as described previously (Chakrabarti et al., 2015).

### Live pseudopupil imaging

Pseudopupil imaging was performed at 16× magnification on flies expressing a single copy of PH-PLCδ–GFP driven by the *trp* promoter. The protocol is as described previously (Chakrabarti et al., 2015). For quantification of recovery, the fluorescence value of image ‘b’ (see Fig. 1Fi) was subtracted from all images and the fluorescence value of image ‘a’ was set as 100. The fluorescence value of subsequent images (i.e. b–i) were normalised to the value of ‘a’.

### Lipid transfer assays for PtdIns, PtdCho and PtdOH

Transfer assays utilised an appropriately radiolabelled donor compartment and an unlabelled acceptor compartment as described previously (Garner et al., 2012). To monitor PtdOH transfer, liposomes (PtdCho:PtdOH, 98:2) containing radiolabelled PtdOH were used as a donor and mitochondria were used as an acceptor. After incubation at 37°C for 30 min in the presence of the transfer proteins, mitochondria were recovered by centrifugation (10,000 ***g*** for 10 min at 4°C). The supernatant was removed and the mitochondrial pellet was resuspended in 500 µl sucrose-EDTA (1 mM EDTA, 0.25 M sucrose). The sample was transferred to a new eppendorf tube and layered on a cushion of 14.3% sucrose solution. After centrifugation of the mitochondria through the sucrose cushion, the pellet was solubilised with 10% SDS and the samples incubated at 95°C for 5 min. The radioactivity in the solubilised samples was monitored by scintillation counting. The PtdOH transfer was expressed as a percentage of input counts in the assay after deducting the background counts.

### Binding of cellular lipids by RDGB proteins

Association of cellular lipids with the RDGB proteins was analysed in HL60 cells exactly as described previously (Ségui et al., 2002; Tilley et al., 2004).

### Reconstitution of G-protein-stimulated PLC activity with PITPs

G-protein stimulated PLC activity was reconstituted exactly as described previously (Cunningham et al., 1995).

### Western blotting

Protein extracts from fly heads were separated by SDS-PAGE and transferred onto nitrocellulose membrane using semi-dry transfer (Chakrabarti et al., 2015). Primary antibodies were against: RDGB (1:4000, polyclonal; made in-house against the PITP domain of RDGB), anti-tubulin (1:4000; E7 DSHB), PITPα (1:1000; raised in-house; Cosker et al., 2008) and TRP (1:5000; made in-house). All secondary antibodies (Jackson Immunochemicals) were used at 1:10,000 dilution.

### Lipid mass spectrometry

Shotgun mass-spectrometry was used to estimate PtdOH and DAG levels in *Drosophila* head extracts. The amount of PtdOH was normalised to total lipid phosphate content. 10 heads per sample (1-day-old flies) were homogenised in 0.1 ml methanol [having 51.98 pmole of (12:0/13:0) PtdOH and 11,140 pmole of (10:0/10:0/0:0) DAG as internal standard] using an automated homogeniser. 0.8 ml chloroform was added and left to stand for 10 min. 0.88% KCl (0.4 ml) was added to split the phases. The organic phase was dried and resuspended in 400 µl of chloroform:methanol (1:2). The total lipid phosphate was quantified from the lipid extract prior to infusion.

Experiments were performed on an LTQ Orbitrap XL (Thermo Fisher Scientific). Stable ESI-based ionisation was achieved using a robotic nanoflow ion source TriVersa NanoMate (Advion BioSciences) using chips with a spraying nozzle diameter of 4.1 μm. Ionisation voltage was 1.2 kV; back pressure was set at 0.95 psi. The temperature of the ion transfer capillary was 180°C. Acquisitions were performed at the mass resolution R*_m_*_/*z*400_=100,000. For analysis, 60 μl of samples were loaded onto the 96-well plate (Eppendorf) of the TriVersa NanoMate ion source. Each sample was infused for 20 min. Lipids were identified with the LipidXplorer software (Herzog et al., 2012) by matching the *m*/*z* of their monoisotopic peaks to the corresponding elemental composition constraints. A Molecular Fragmentation Query Language (MFQL) file was compiled for PtdOH and DAG. Mass tolerance was 10 ppm and the intensity threshold was set according to the noise level reported by Xcalibur software (Thermo Scientific).

### Cell fractionation

Fly heads (from ∼500 flies) were homogenised in lysis buffer (pH 7.4) (20 mM HEPES, 30 mM NaCl, protease inhibitor) and centrifuged at 166,000 ***g*** in Beckman TLA55 rotor for 30 min to separate membrane from cytosol. The membrane pellet was washed with lysis buffer (without EDTA) and resuspended in the same. Aliquots of membrane fraction were treated with following reagents on ice for 1 h: lysis buffer (control), 1 M NaCl, 0.2 M Na_2_CO_3_ pH 11, 6 M guanidine chloride (denaturant) and 2% Triton X-100. Post treatment, each sample was centrifuged at 172,000 ***g*** and supernatant was removed. The pellet was mixed with protein lysis buffer and run on a polyacrylamide gel.

## Supplementary Material

Supplementary Material
