# Supplementary material for: RDGBα, a PtdIns-PtdOH transfer protein, regulates G-protein-coupled PtdIns(4,5)P2 signalling during Drosophila phototransduction
Source: J Cell Sci. 2015 Sep 1;128(17):3330–44. doi: 10.1242/jcs.173476 (PMC4582195; doi:10.1242/jcs.173476)
Supplement: Supplementary Material [file supp_128_17_3330__index.html]

RDGBα, a PtdIns-PtdOH transfer protein, regulates G-protein-coupled PtdIns(4,5)P2 signalling during Drosophila phototransduction — Supplementary Material 

# RDGBα, a PtdIns-PtdOH transfer protein, regulates G-protein-coupled PtdIns(4,5)*P*2 signalling during *Drosophila* phototransduction

## JCS173476 Supplementary Material

- Supplementary Material
