# Supplementary material for: RDGBα, a PtdIns-PtdOH transfer protein, regulates G-protein-coupled PtdIns(4,5)P2 signalling during Drosophila phototransduction
Source: J Cell Sci. 2015 Sep 1;128(17):3330–44. doi: 10.1242/jcs.173476 (PMC4582195; doi:10.1242/jcs.173476)
Supplement: Supplementary Material [file supp_jcs.173476_JCS173476supp.pdf]

Supplementary data Yadav et.al

Supplementary Figures

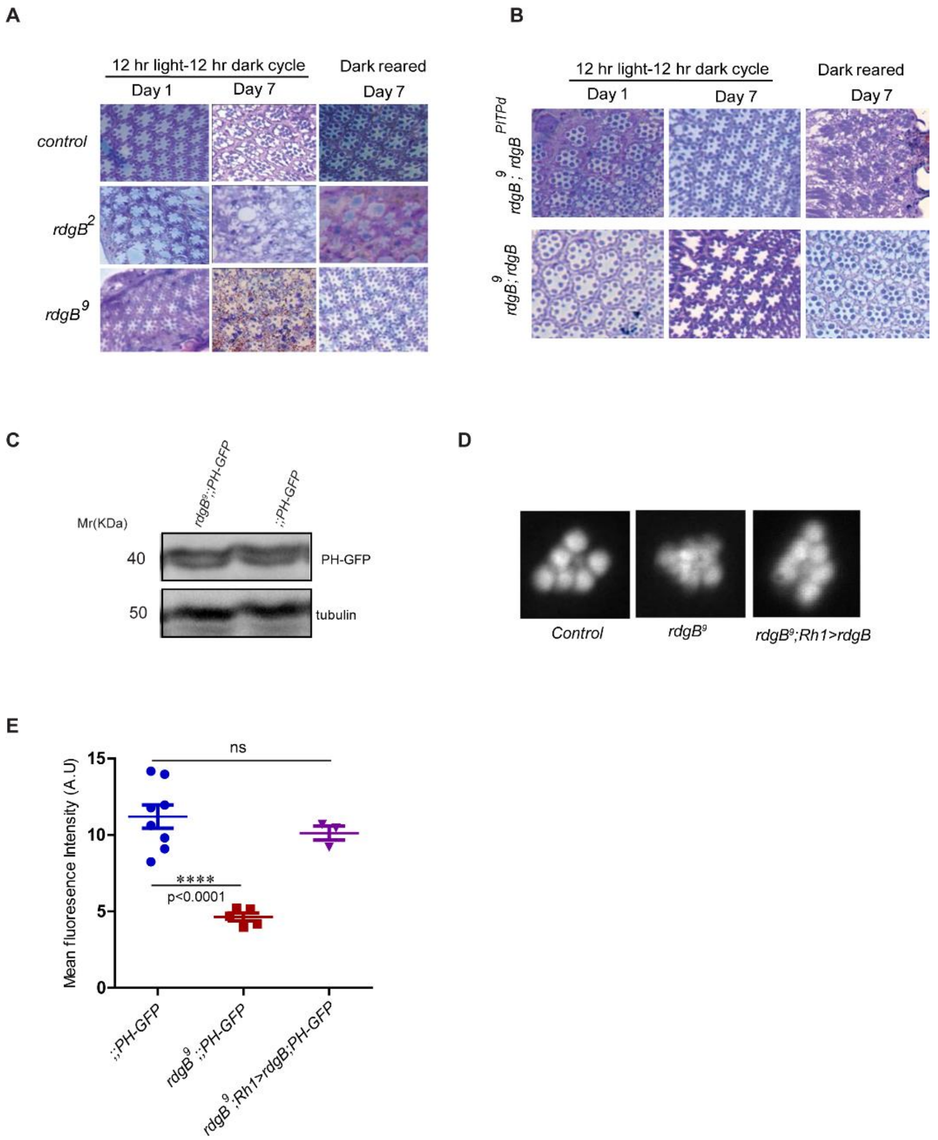

Figure S1

A,B) Representative plastic section images of 1 and 7 days old flies showing extent of retinal degeneration. Genotypes are indicated to the left of the panels and the illumination conditions used are shown above the panels. C) Western blot of head extracts made from wild type and *rdgB*^{9} flies expressing PH-PLCδ::GFP, showing expression of probe. Blot was re-probed with anti-tubulin antibody as a loading control. Molecular weight and the antibody used are marked on the sides. D) Representative first image acquired during fluorescent pseudopupil imaging of indicated genotypes. All flies were one day old.

E) Quantification of mean fluorescence intensity of the first image captured during pseudopupil imaging of flies of mentioned genotypes.

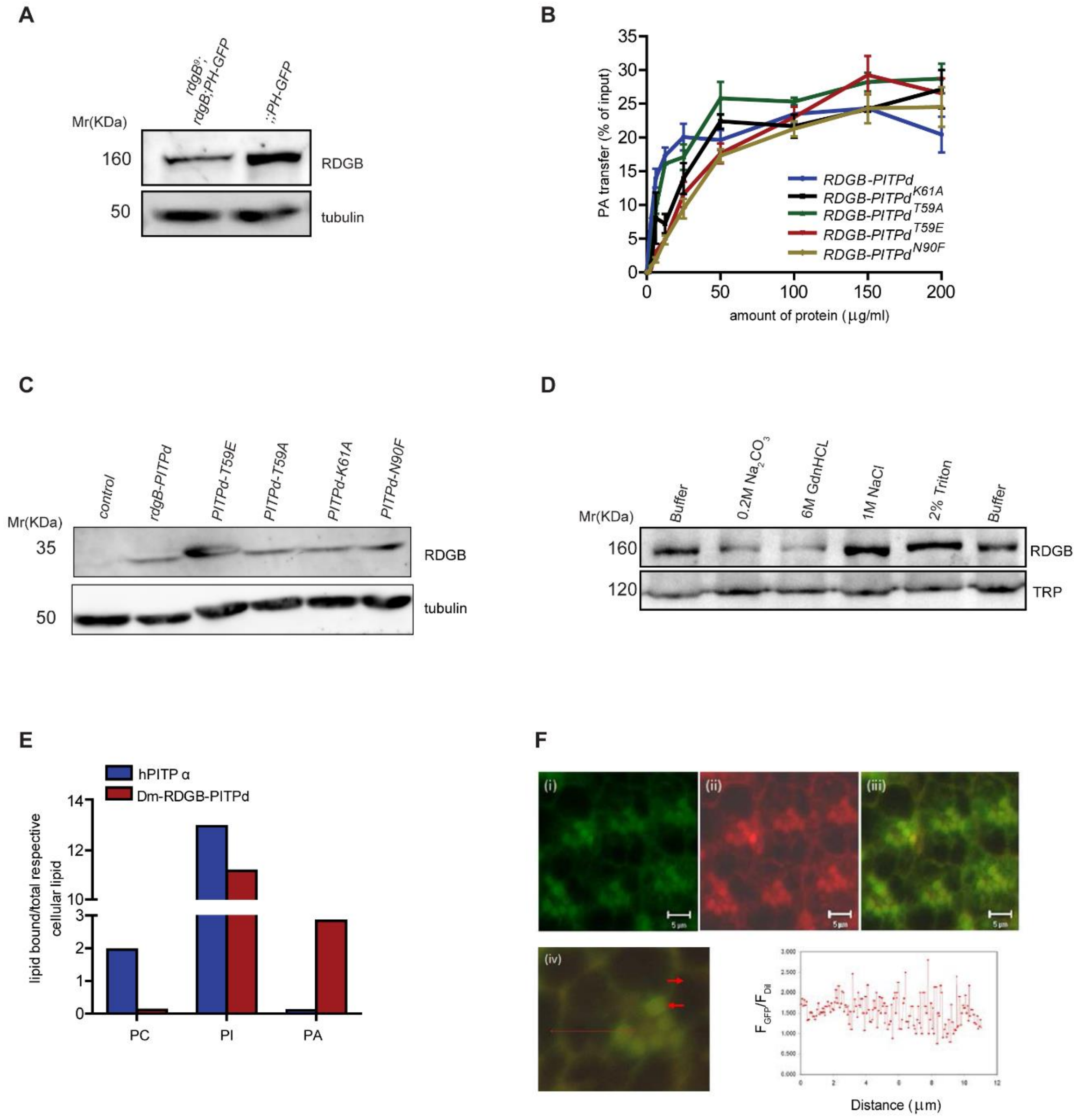

Figure S2

A) Western blot of head extracts made from flies of mentioned genotype, showing expression of RDGB. B) PtdOH transfer activity of RDGB-PITPd and PIBR mutant recombinant proteins. Transfer activity for PtdOH is shown for increasing amounts of recombinant protein used. Y axis represents amount of lipid as a percentage of total input count. Error bars represent S.D. C) Western blot of head extracts made from flies of mentioned genotype, showing expression of PIBR mutant versions of the PITPd of RDGB. Upper labels mark the mutation in the PITP domain. Blot was re-probed with anti-tubulin antibody as a loading control. Molecular weight and the antibody used are marked on the sides. D) Western blot of membrane fraction of head extract from wild type flies. Head extract from ca.500 flies was centrifuged at 52K rpm to separate

membranes from cytosol. The membrane fraction was subjected to mentioned treatments [0.2M Na_2CO_3 (pH=11); 6MGdHCl (denaturant)] and re-subjected to ultra centrifugation at 53K rpm. Sample buffer was added to the washed pellet and was loaded onto SDS gel. Gel was re-probed with anti-trp, an integral membrane protein which is not affected. E) Comparison of lipid binding to Dm-RDGB^{PITPd} and hPITPα: The individual lipid bound to the PITP is expressed as a fraction of the respective lipid available in the cells providing a measure of affinity for that lipid. F) Confocal image of the transverse section of photoreceptors expressing the PH domain of PLCδ fused to GFP (PH-PLCδ::GFP). (i) The PH- PLCδ::GFP probe is expressed on the plasma membrane of the photoreceptor (ii) The same photoreceptor stained with a generic fluorescent membrane dye DiI [1,1'-dioctadecyl-3,3,3'3'- tetramethylindocarbocyanine perchlorate].

(iii) Overlay image of both PH-PLCδ::GFP and DiI fluorescence. (iv) Magnified view of a single ommatidium showing the overlay image. Apical (left headed arrow) and basolateral (right headed arrow) membrane of the photoreceptor are shown. A red line drawn across the photoreceptor is shown and the Ratio of the PH-PLCδ::GFP fluorescence (F_{GFP}) to DiI (F_{DiI}) fluorescence is shown

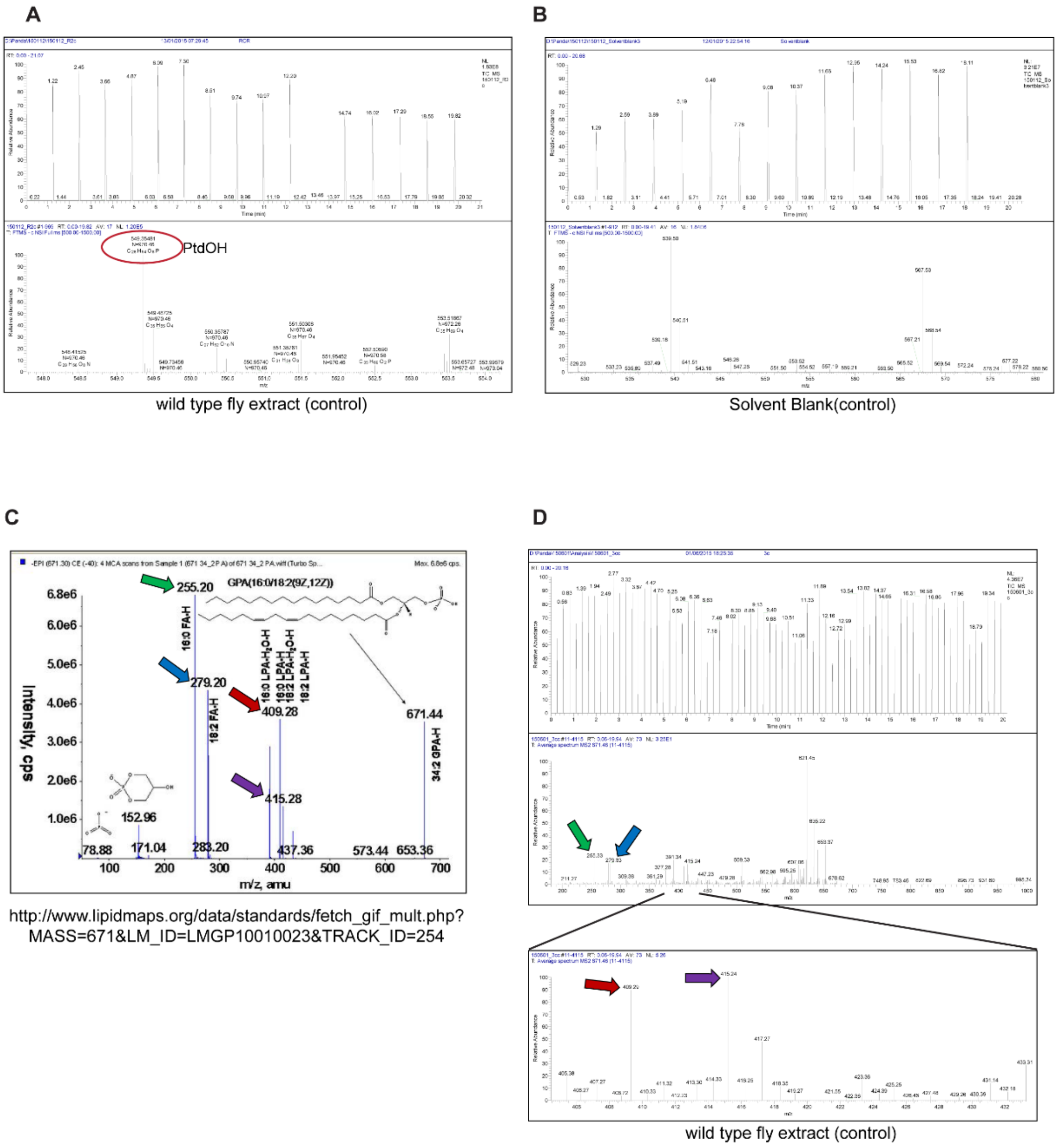

Figure S3

A) Total ion chromatogram from a MS acquisition of wildtype lipid extract. The MS annotation of 549.35481 is PtdOH(12:0/13:0) and was used as internal standard. B) The total ion chromatogram from a MS acquisition of the solvent blank where the m/z at 549.35481 is missing. C) The MS/MS fragmentation pattern of PtdOH (34:2), reported in lipidmaps consortium. D) MS/MS fragmentation pattern of 671.46 (analysed as PtdOH(34:2)) from wildtype lipid extract . The coloured arrows indicate matched MS/MS fragmentation, which further confirms the annotation.

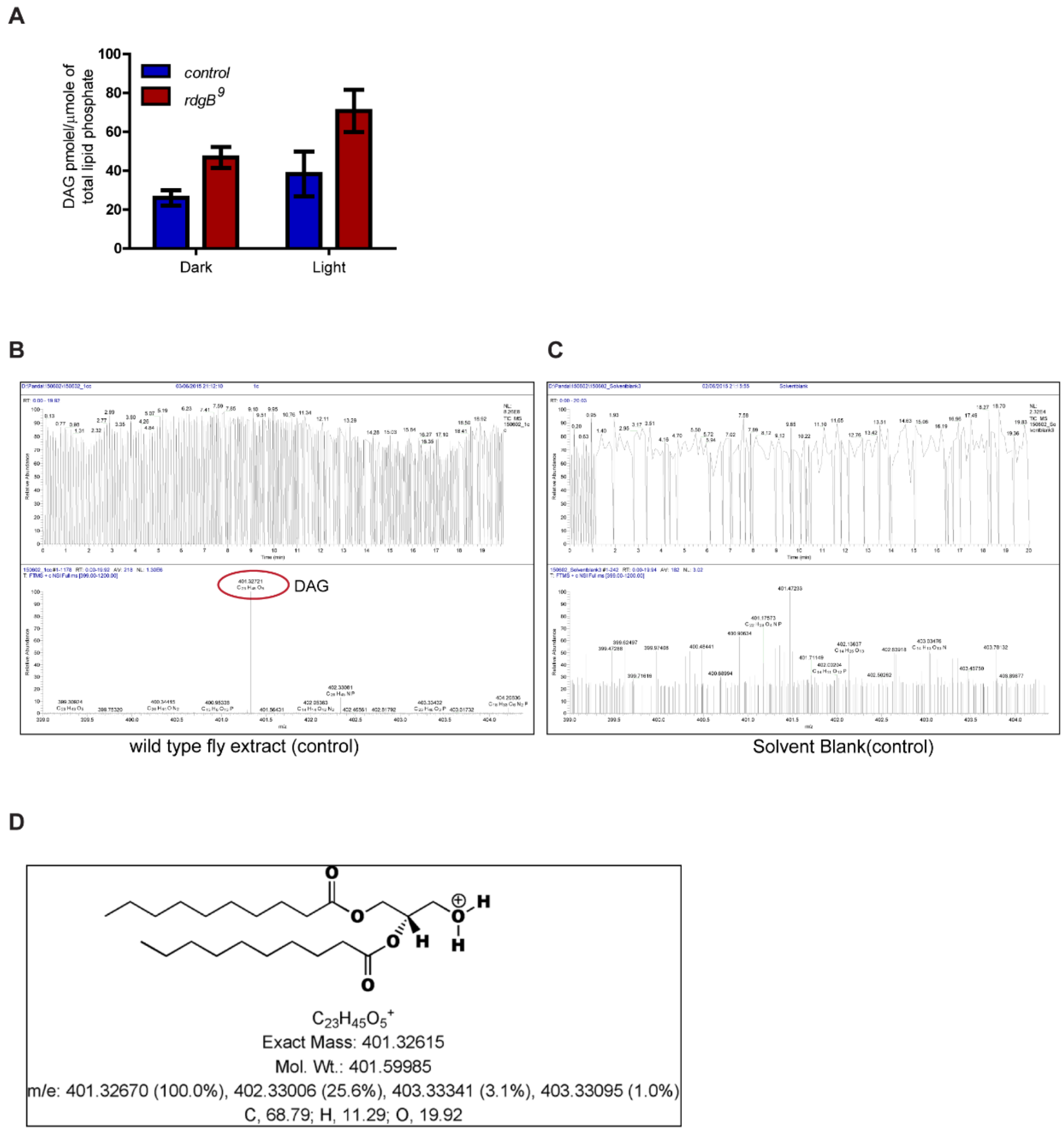

Figure S4

A) Measurement of total DAG levels in heads of wild type and *rdgB*^{9} flies reared in dark and after 1 minute of bright light stimulation. DAG levels were normalized to the lipid phosphate content of sample. Values represent mean ± S.E.M. B) Total ion chromatogram from an MS acquisition of wildtype lipid extract. The MS annotation of 401.59985 is DAG (10:0/10:0/0:0) and was used as internal standard. C) The total ion chromatogram from a MS acquisition of the solvent blank where the m/z at 401.59985 is missing. D) Chemical structure of DAG (10:0/10:0/0:0) with its elemental composition and molecular mass.
